# The use of amino acid‐based nutritional feeds is effective in the dietary management of pediatric eosinophilic oesophagitis

**DOI:** 10.1002/iid3.273

**Published:** 2019-11-06

**Authors:** Kiranjit Atwal, Gary P. Hubbard, Carina Venter, Rebecca J. Stratton

**Affiliations:** ^1^ Medical Affairs Nutricia Ltd Trowbridge United Kingdom; ^2^ Section of Allergy and Immunology, Children's Hospital Colorado University of Colorado Denver School of Medicine Colorado; ^3^ Faculty of Medicine University of Southampton Southampton United Kingdom

**Keywords:** elemental diet, pediatric eosinophilic oesophagitis, remission

## Abstract

**Introduction:**

Eosinophilic oesophagitis (EoE) is an immune‐mediated, chronic disease characterized by eosinophilic inflammation and esophageal dysfunction. Specific food allergens including cow's milk protein, are partially causative to disease progression, and dietary management forms three main options; the elemental diet (ED), the empirical elimination diet (EED), and the targeted elimination diet (TED). The dietary choice should be individualized, however, the European Society for Pediatric Gastroenterology, Hepatology and Nutrition guidelines recommend an ED for pediatric EoE with multiple food allergies, failure to thrive, unresponsive disease or unable to follow a highly restricted diet. The aim of this narrative review was to explore the effectiveness of the ED (using amino acid formula [AAF]), in the management of pediatric EoE.

**Methods:**

Literature searches were performed to identify eligible studies that described outcomes including eosinophil count, clinical symptoms, growth, and medications.

**Results:**

Overall, 10 eligible studies were found, with n = 462 patients assigned to receive AAF from a total of n = 748 (average age 6.7 years), for a duration of 4 to 8 weeks. The use of AAF reduced eosinophil levels and demonstrated remission (defined as ≤10 eosinophils per high power field) in 75%‐100% of children with improvements, if not resolution, in clinical symptoms. AAF was more clinically effective than the use of the EED or TED, where remission rates were 75%‐81% and 40%‐69%, respectively. Few studies collected growth outcomes, however where documented these were positive for those on AAF. The long‐term impacts of each diet were not thoroughly explored.

**Conclusions:**

The use of AAF is a clinically effective management option for pediatric EoE, and further research is required to guide long‐term management.

## 1 INTRODUCTION

Eosinophilic oesophagitis (EoE) is an immune‐mediated, chronic disease of the esophagus, characterized by dysfunction and eosinophilic inflammation.^1^ Clinical features vary according to age; infants and toddlers typically present with nonspecific symptoms such as feeding difficulties, vomiting, regurgitation, and food aversion, leading to failure to thrive if misdiagnosed and mismanaged.^2^, ^3^ Young children often experience vomiting and abdominal pain, whereas older children and teenagers experience gastroesophageal reflux disease, dysphagia, and food impaction.^2^, ^3^ On the basis of American and European consensus, diagnosis of EoE is confirmed by at least one or more biopsies in the proximal or distal esophagus showing ≥15 eosinophils per high power microscopic field (hpf) per biopsy specimen. Endoscopy may show evidence of esophageal rings with thickened, pale mucosa, white exudate and linear furrows which are graded by severity of inflammation.^1^, ^2^, ^4^

The inflammatory process of EoE involves T‐helper type 2 cells which are activated in response to offending allergens, leading to a cascading chain of events involving proliferation and maturation of eosinophils which target the epithelial barrier of the esophagus.^5^, ^6^ This leads to esophageal dysfunction, inflammation, and fibrosis in the esophagus by the deposition of collagen.^5^ Elevated specific immunoglobulin E (IgE) antibodies and IgE sensitization to allergens found in food such as cow's milk and wheat, have been associated concurrently with EoE.^7^, ^8^, ^9^ However, as EoE is a non‐IgE‐mediated presentation of allergic disease, guidelines do not recommend IgE testing such as skin prick tests (SPTs) which may lead to unreliable results and unnecessary food avoidance.^10^, ^11^

Variation exists in the literature on the prevalence of pediatric EoE where it is reported, varying between 0.2 and 43 cases per 100 000.^12^ A recent meta‐analysis of population‐based studies published pooled the prevalence of pediatric EoE across North America and Europe to estimate overall prevalence at 34.4/100 000 and overall incidence at 6.6/100 000.^13^ An increasing trend over the past decade has been captured in several reports; a recent cohort study in Utah found 24 cases/100 000.^14^ Together with this study from Utah, figures for the epidemiology of EoE in European children were simultaneously provided by Arias et al^15^ where the incidence was reported as 10.6/100 000 in children. However, it is not clear whether the incidence, which has increased over the years reflects a true incidence related to environmental factors or the evolution of diagnostics.

Both the American College of Gastroenterology (ACG)^4^ and the European Society for Pediatric Gastroenterology, Hepatology and Nutrition (ESPGHAN)^2^ guidelines for the management of EoE describe three management options, pharmacotherapy, dietary therapy, and endoscopic guided esophageal dilatation.^2^, ^4^, ^5^ Dietary therapy for the management of pediatric EoE takes three forms the elemental diet (ED); targeted elimination diet (TED); and the empirical elimination diet (EED); each of which removes dietary allergens by various means. Guidelines suggest the choice of dietary intervention should be individualized to the patient circumstances, led by an Allergist and Gastroenterologist with careful dietary management by a Registered Dietitian.^2^, ^4^ The ED, using amino acid formula (AAF) is recommended by several guidelines.^2^, ^4^ AAF provides free amino acids, void of intact proteins and peptides, and when used exclusively, provides nil exposure to dietary allergens. ESPGHAN guidelines recommend that an ED is followed for 4 weeks, particularly in children with multiple food allergies, growth faltering, unresponsive disease, and highly restricted diets.^2^ However, more recent guidelines have suggested that an ED should only be considered after failure of medical treatment and/or elimination diets.^10^ ESPGHAN recommends the TED is followed for 8 to 12 weeks, which involves the exclusion of foods based on results of specific IgE tests, including SPTs and atopy patch tests (APTs).^2^, ^4^ However, the specificity of the TED is poor, less commonly used and recent guidelines do not endorse its use.^10^, ^16^ ESPGHAN recommends the EED is followed for 8 to 12 weeks which involves the exclusion of six common food containing allergens: cow's milk, wheat, soy, egg, peanuts, and fish/shellfish.^2^ This diet is more widely followed in practice with greater success and recent guidelines favor its use compared with TED and ED.^10^ Results from a European pediatric EoE cohort review recently demonstrated by process of elimination and food challenge, that cow's milk, egg, wheat/gluten, and peanut were foods most commonly found to be triggers of EoE (42%, 21.5%, 10.9%, and 9.9%, respectively).^17^ Furthermore, results of elimination diets that sequentially exclude these foods in groups of two, before eliminating all six foods, known as the “step‐up” diet, have demonstrated promising remission rates (43% in children), and are becoming common in practice.^16^, ^18^

Many guidelines that define EoE fail to define histological remission by the number of eosinophils/hpf, although ACG guidelines state that endpoints of EoE include improvements in histology which could imply <15 eosinophils/hpf is successful.^2^, ^4^, ^10^ A systematic review found most studies used <5 eosinophils/hpf as criteria for histological remission, although some classed histological improvement up to ≤15 eosinophils/hpf, demonstrating a large variation.^19^ It is important to note that symptom resolution, which is readily assessed, is an important parameter in treatment success and needs to be considered in conjunction with histology.^4^ A new framework for treatment response in EoE has recently proposed histology, symptom and endoscopy parameters for three categories of EoE resolution; nonresponse, response, and complete normalization.^20^

Whilst there are reviews of the literature on the effectiveness of the various dietary options in pediatric EoE,^21^, ^22^, ^23^ there is little analysis on the effectiveness of the ED (using AAF) in pediatric EoE. The aim of this narrative review was therefore to explore the effectiveness of the use of AAF in the management of pediatric EoE.

## 2 MATERIALS AND METHODS

Relevant studies were identified by searching electronic databases that were last accessed last on 1 May 2019. The databases searched included PubMed,^24^ Google Scholar,^25^ Dialog, ISI Web of Knowledge,^26^ The Cochrane Library,^27^ BMJ Clinical Evidence,^28^ NHS Evidence,^29^ Turning Research into Practice,^30^ and CINAHL.^31^ Due to the restricted scope of the review, the search was initially broad, focussing on studies of patients including the use of AAF, using the search terms “elemental” and the brand names of leading AAFs available in western countries. Studies were then identified that had specifically investigated the use of AAFs for the dietary management of pediatric EoE. Bibliographies of identified trials, related reviews and conference proceedings were also analyzed, and experts were consulted for any additional studies.

### 2.1 Study selection criteria

Studies available as full papers or abstracts were deemed eligible if they met the inclusion criteria. All study types including pediatric patients (<18 years) with diagnosed EoE, where AAFs were used (randomized and nonrandomized trials, retrospective chart reviews, etc) in which patient demographics, dietary intervention, and histological/symptomatic outcomes were reported, qualified for the review. Any studies which compared the use of AAF were included, as were studies comparing the ED to the TED, EED or other standard practice. Single case reports, consensus statements, review articles without original data, abstracts/posters without sufficient detail, articles with duplicated data, and articles in non‐English language were all excluded.

### 2.2 Data extraction and outcome measures

Data extracted from study manuscripts included, study design, mean patient age (years), number of patients receiving different interventions, intervention details, follow‐up period (months), biopsy (reported as per hpf), measurements of eosinophil counts pre and post‐intervention, % remission rates (as defined by the study authors), clinical symptom resolution (as defined by the study authors, typically gastrointestinal, respiratory, cutaneous, dysphagia), growth parameters (including weight, height, body mass index, and *z*‐scores), and recorded changes in medications. The selected outcome measures in this review are as follows: histology (eosinophilic count), clinical symptoms, growth, and medication. Each outcome measure has been compared by interventions (a) ED only, (b) ED vs TED, (c) ED vs EED, (d) ED vs TED and EED, (e) combination intervention of ED, EED, and TED.

### 2.3 Quality analysis

Eligible studies were assessed for level of quality using the risk of bias in nonrandomized studies of interventions (ROBINS‐I) tool developed by Cochrane^32^ as no randomized trials were identified. The risk of bias was independently assessed by two of the authors and differences resolved by discussion with a third‐party reviewer.

## 3 RESULTS

**Figure 1:**
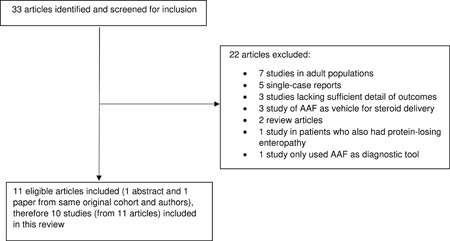
Flowchart of studies identified included and excluded for this literature review

A total of 33 articles were identified from the initial search strategy, which was assessed for eligibility for inclusion in this review (Figure 1). The following were excluded from the review n = 7 studies were in adults (>18 years)^33^, ^34^, ^35^, ^36^, ^37^, ^38^, ^39^; n = 5 articles were single case reports only^40^, ^41^, ^42^, ^43^, ^44^; n = 3 articles mentioned the use of AAF but did not provide sufficient details on the intervention or outcomes^3^, ^45^, ^46^; n = 2 were review papers^23^, ^47^; n = 1 used AAF to confirm food sensitivity outside the context of EoE^48^; n = 1 included patients with eosinophilic gastroenteritis with protein‐losing enteropathy^49^; n = 3 examined the use of a spoon‐fed yogurt‐type amino acid‐based feed as a vehicle for topical steroid delivery.^50^, ^51^, ^52^ One abstract was identified (n = 13 patients) by the same authors of another study on the same cohort (n = 10); however, n = 3 of patients from the abstract were excluded. Data from both articles were reviewed and included in this review but only counted as one study.^53^, ^54^ Overall, 10 studies (from 11 articles) were identified that fully met the selection criteria (Table 1).^53^, ^54^, ^55^, ^56^, ^57^, ^58^, ^59^, ^60^, ^61^, ^62^, ^63^

**Table 1:** Summary of characteristics and overall outcomes from each study

Author	Study design	Risk of bias	Total no. of patients	Age, y	Brand name AAF	Duration on diet, wk	No. of patients on ED	Route (OI/NGT)	Solids permitted	Prediet EoE count/hpf	Postdiet EoE count/hpf	% remission postdiet
Kelly et al^53^	Quasi‐experimental prospective study	Serious	13	6.5^a^	Neocate	6‐8	13	NGT	…	ED: 32	ED: 2	…
Kelly et al^54^	Quasi‐experimental prospective study	Serious	10	5.2^a^	Neocate	6	10	OI (n = 2)	Yes (corn and apple only)	ED: 41^a^	ED: 0.5^a^	…
								NGT (n = 4)				
								OI+NGT (n = 4)				
Markowitz et al^55^	Quasi‐experimental prospective study	Low	51	8.3^b^	Neocate	4	51	OI (n = 3)	Yes (water, apple, or grapes)	ED: 33.7^a^	ED: 1^a^	…
								NGT (n = 48)				
Rizo Pascual et al^56^	Quasi‐experimental prospective study	Critical	14	9^a^	…	8	3	…	…	ED: >20	ED: <10	ED: 100%^c^
										TED: >20	TED: >10	TED: 42%^c^
Spergel et al^57^	Quasi‐experimental cohort study	Serious	146	6.5^a^	Neocate	6	39	OI	…	ED: 14.2‐48.4^b^	ED: 0.5‐1.1^b^	ED: 98%^d^
					Elecare			NGT		TED: 48.4^b^	TED: <5^b^	TED: 88%^d^
Al‐Hussaini et al^58^	Retrospective cohort study	Critical	14	6^a^	Neocate	8	4	OI	…	ED: >15	ED: <5	ED: 75%^e^
										TED: >15	TED: 14‐5	TED: 40%^e^
Liacouras et al^62^	Retrospective cohort study	Serious	247	9.1^a^	Neocate	4	172	OI (n = 35)	Yes (white grapes, and apples)	ED: 38.7^b^	ED: 1.1^b^	…
					Elecare			NGT (n = 137)		TED: 47.5^b^	TED: 5.3^b^	…
Kagalwalla et al^59^	Retrospective cohort study	Low	60	6^a^	Neocate	6	25	OI (n = 7)	No	ED: 58.8	ED: 3.7	ED: 88%^f^
					Elecare			NGT (n = 9)				
								GT (n = 9)		EED: 80.2	EED: 13.6	EED: 74%^f^
Henderson et al^60^	Retrospective cohort study	Moderate	98	5.9^a^	Neocate	18	49	OI (n = 27)	No	ED: 51^a^	ED: 1^a^	ED: 96%^g^
					Elecare			NGT (n = 22)				
										TED: 38^a^	TED: 7^a^	TED: 65%^g^
										EED: 76.5^a^	EED: 2.5^a^	EED: 81%^g^
Colson et al^61^	Retrospective cohort review	Serious	59	6^a^	Puramino	8	59	…	…	30^a^	5^a^	59%
					Neocate							
Kalach et al^63^	Retrospective cohort review	…	49	…	Neocate	12	49			>15	<5	53%

Abbreviations: ED, elemental diet; EED, empirical elimination diet; EoE, eosinophilic oesophagitis; n, number of subjects; no., number; NGT, nasogastric tube; OI, oral intake; TED, targeted elimination diet; y, years.

^a^ Median.

^b^ Mean.

^c^ Remission was defined as <10/hpf.

^d^ Remission was defined as <5/hpf.

^e^ Remission was defined as <5/hpf and asymptomatic.

^f^ Remission was defined as <10/hpf.

^g^ Remission was defined as  <15/hpf.

Of the included studies, the majority (n = 6) were retrospective design (n = 5 retrospective cohort studies^58^, ^60^, ^61^, ^62^, ^63^ and n = 1 retrospective observational study 59), n = 3 quasi‐experimental prospective studies^53^, ^54^, ^55^, ^56^ and n = 1 a quasi‐experimental cohort study^57^ (Table 1). The risk of bias was determined in all studies n = 2 low risk of bias,^55^, ^59^ n = 1 moderate risk of bias,^60^ n = 4 serious risk of bias,^54^, ^57^, ^61^, ^62^ n = 2 critical risk of bias,^56^, ^58^ n = 1 studies had insufficient information to form an assessment of bias^63^ (Table 1). Only n = 2 studies solely investigated the ED^53^, ^54^, ^55^ with the remainder comparing the ED to the TED (n = 4),^56^, ^57^, ^58^, ^62^ the ED to the EED (n = 1),^59^ the ED to both the EED and TED (n = 1)^60^ and n = 2 studies combined all three diets into a single intervention.^61^, ^63^ Across all studies the number of patients who received AAF was n = 462, out of a total sample size of n = 748 and mean age was 6.7 years (range, 4 months‐20 years). Intervention duration ranged from 4 to 8 weeks where specified and mean follow‐up with repeat endoscopies varied between 1 and 9 months (Table 1). In n = 7 studies, AAF was initiated due to treatment failure of proton pump inhibitors (PPIs) over a duration of 4 to 8 weeks.^53^, ^54^, ^55^, ^57^, ^59^, ^60^, ^61^, ^62^

### 3.1 Overall summary of outcomes

Overall, the data extracted from eligible studies on the use of AAF in pediatric EoE included in this narrative review demonstrated that the ED is a clinically effective management option for the induction of EoE remission. The ED resulted in remission (defined as ≤10 eosinophils/hpf) in 75%‐100% of children (n = 5 studies^56^, ^57^, ^58^, ^59^, ^60^) and improvements or resolution of clinical symptoms in the majority of patients (n = 5 studies^53^, ^54^, ^55^, ^57^, ^59^, ^61^), which is comparable to other data reported.^21^

In the studies that compared the diets, the ED was more clinically effective than the EED or TED, where remission rates were 75%‐81% (n = 2 studies^59^, ^60^), and 40%‐88% (n = 4 studies^56^, ^57^, ^58^, ^60^), respectively. This was particularly demonstrated in the studies by Kagalwalla et al^59^ and Spergel et al^57^ where AAF use successfully resolved eosinophilic infiltration in 95% of patients after failure of the previously assigned interventions of EED or TED. The ED alone also appeared more effective than when used in combination with other diets, such as the TED, where remission was between 53%‐59% (n = 2 studies^61^, ^63^).

Although all dietary intervention time ranged between 4 to 8 weeks, clinical response to AAF use was seen as rapidly as 8.5 days (±3.8) in one study.^55^ No study used ED as a treatment option for more than 18 weeks. In comparison, rapid observations were not documented by any studies that examined the EED and TED. The ED resulted in improvements in growth (n = 4 studies^54^, ^59^, ^61^, ^63^), reduced medication (n = 1 cohort^53^, ^54^) and was also observed to resolve long‐term symptoms in most patients which included food aversion, abdominal pain, vomiting, diarrhea, dysphagia, heartburn, mucosal healing and chest pain, when compared with EED and TED.

### 3.2 Histology (eosinophil count)

#### 3.2.1 ED only trials

**Figure 2:**
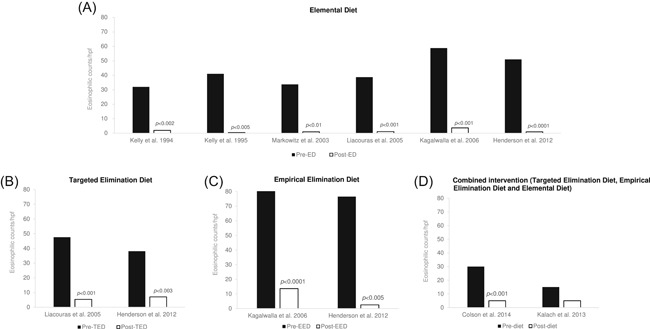
Summary of histological (eosinophilic) change pre and post dietary intervention by dietary intervention type from each study (A) elemental diet (n = 5 studies); (B) targeted elimination diet (n = 2); (C) empirical elimination diet (n = 2 studies); (D) combined intervention including targeted elimination diet, empirical elimination diet and element diet (n = 2 studies). Histological change measured by eosinophilic counts per high power field (hpf) from the biopsy. *P* values are based on significant differences in eosinophilic counts between pre and postdiet within each study only

The earliest cohort reported in the literature was by Kelly et al in 1994^53^ and again in 1995,^54^ whereby children with eosinophilic infiltration of the esophagus and chronic reflux symptoms (who failed standard PPI therapy), were exclusively fed AAF. In the 1994 analysis, n = 13 (median age, 6.5 years; range, 2‐15 years) were followed up after 6 to 8 weeks and demonstrated significant reductions in mean eosinophils: 32 to 2/hpf at follow‐up (*P* < .002) (Figure 2A). In the 1995^54^ analysis of the cohort, where n = 3 were excluded on the basis of normal prediet biopsies, n = 10 (median age, 5 years; range, 8 months‐12.5 years) were analyzed and after a median of 17 weeks on AAF, median eosinophils significantly dropped from 41/hpf (range, 15‐100) to 0.5/hpf (range, 0‐22) (*P* < .005) at follow‐up (Figure 2A). Neither definition of histological remission or the rates of patients’ remission were outlined. Treatment failures described in the cohort before initiating AAF included prescription of prokinetics and Nissan's fundoplication. This cohort formed the preliminary beginnings of research into the role of dietary allergens in EoE.

In a larger prospective study by Markowitz et al 2003,^55^ children and adolescents with EoE (n = 51; mean age, 8.3 years; range, 3‐16 years) who failed standard PPI therapy, were placed on AAF exclusively for 1 month. Median esophageal eosinophils reduced significantly from 33.7 to 1.0/hpf (*P* < .01) (Figure 2A) after an average of 8.5 days (±3.8). Overall, 96% of patients (n = 49) responded to the use of AAF but rates of histological remission, or at which level it was defined were not described.

#### 3.2.2 ED vs TED trials

Four of the studies included in this review conducted comparative double‐arm trials using the ED and TED. Rizo Pascual et al 2011^56^ conducted a prospective comparative study on children with EoE (n = 15; mean age, 9 years; range, 2 years 8 months‐14 years 5 months), and 100% of AAF users (n = 3) demonstrated histological remission (defined as <10 eosinophils/hpf) after 2 months. In contrast, those on the TED (n = 12) had lower success with 50% (n = 6) failing to respond, one drop‐out and only 42% (n = 5) achieving remission after 2 months. One patient who had failed the TED later started AAF and achieved histological remission.

Spergel et al 2005^57^ compared the effectiveness of AAF and the TED in children and adolescents with EoE (n = 146; mean age, 6.5 years; range, 4 months‐20 years) in a retrospective cohort study. Results from APTs and SPTs guided eliminations in the TED; however, if more than 10 food allergens were positively identified, AAF was initiated. Initially n = 120 were started on the TED and n = 26 on AAF for a minimum of 6 weeks; however, n = 14 of those on the TED demonstrated incomplete histological improvement and transitioned to AAF (total on AAF n = 40). Complete resolution was observed in 98% of those on AAF compared with only 88% of those on the TED. The “responder group” (defined as <5 eosinophils/hpf) demonstrated mean eosinophilic reductions from 48.4 to 1.1/hpf at follow‐up (n = 112) and of those on AAF, 98% were responders (n = 39) whereas only 61% on the TED (n = 73) were responders. Of the patients who did not initially respond to the TED (n = 14, mean eosinophils 14.2/hpf), switch to AAF resulted in a mean lowering of eosinophils to 0.5/hpf. The single patient who did not tolerate AAF went on to develop complications (eosinophilic gastroenteritis).

Al‐Hussaini et al 2013^58^ retrospectively analyzed a small cohort of children with EoE (mean age, 6 years; range, 1‐11 years) comparing the ED (n = 4) and TED (n = 10) and found remission (defined as ≤5 eosinophils/hpf) in 75% of patients using AAF after 2 months. Of the children on the TED, 40% had partial remission (defined as 5‐14 eosinophils/hpf) and the remaining were nonresponsive (eosinophils remained ≥15/hpf).

A large retrospective cohort study over 10 years by Liacouras et al 2005,^62^ compared children (mean age, 10.4 years) with EoE managed by either AAF (n = 172) or TED (n = 132) and found that 93% of children on AAF (n = 160) had significant histological improvement. Mean eosinophils reduced from 38.7 to 1.1/hpf (*P* < .001) over a mean time of 9 months (Figure 2A), whereas of those on the TED, only 57% (n = 75) achieved histological improvement, where mean eosinophils reduced from 47.5 to 5.3/hpf (*P* < .001*)* (Figure 2B). Residual eosinophil counts were significantly lower in the AAF group vs the TED group at follow‐up (1.1 vs 5.3/hpf, respectively; *P < *.05). Most patients receiving AAF (77%; n = 134) discontinued after a mean time of 5.3 months (range, 3‐18 months). Of these patients, after 63 months from initial histological resolution, only n = 3 were able to ingest a food previously known to be related to their EoE.

#### 3.2.3 ED vs EED trials

The only trial to compare the effectiveness of ED and EED was by Kagalwalla et al 2006^59^ (Table 1) in a comparative retrospective study of n = 60 children with EoE (mean age, 6 years). In the AAF group (n = 25) mean eosinophils reduced significantly from 58.8 to 3.6/hpf (*P* < .001) (Figure 2A) 6 weeks postdiet. Posttreatment biopsies in 88% of these patients met the criteria for significant histological improvement (≤10 eosinophils/hpf). Improvements were also seen in the EED group (n = 35) but not to the same extent, with mean eosinophils reducing from 80.2 to 13.6/hpf (*P* < .0001) (Figure 2C) and only 74% meeting the criteria for significant histological improvement. Of the n = 6 patients who did not respond to the EED (mean eosinophils 58/hpf), n = 5 went onto AAF, where n = 3 developed significant histological improvements (≤10 eosinophils/hpf) and n = 1 demonstrated partial histological improvement (≤20 eosinophils/hpf).

#### 3.2.4 ED vs TED and EED trials

The only study to compare the effectiveness of all three dietary interventions in children with EoE was led by Henderson et al 2012.^60^ The use of AAF (n = 49), the EED (n = 26) and the TED (n = 23) was compared in n = 98 children (mean age, 5.9 years). With the use of AAF, median eosinophils dropped significantly from 51 to 1/hpf (*P *< .0001) (Figure 2A) and were significantly greater than the TED (*P *< .01), although there was no significant difference between the EED and AAF, nor between the EED and the TED. Significant reductions in postdiet eosinophils were demonstrated across all three diets (Figure 2). Remission (defined as <15 eosinophils/hpf) was obtained in 96% of those on AAF, 81% of those on the EED and 65% of those on the TED group. The AAF group showed a higher complete remission rate (defined as ≤1 eosinophils/hpf) of 59% (*P *= .04) and lower nonremission (defined as ≥15 eosinophils/hpf) of 4% (*P *= .001) compared with the TED group. The odds of postdiet remission were reported for those on AAF as 5.6‐fold greater compared with EED, and 12.5‐fold greater than the TED (no difference between the EED and TED).

#### 3.2.5 Combination intervention (ED, EED, and TED) trials

Two studies combined all three dietary interventions into a single intervention (Figure 2D); Colson et al 2014^61^ carried out a retrospective cohort review in n = 59 children with EoE (median age, 6.5 years; range, 9 months‐16 years) where all children initiated EED combined with eliminations of foods with a positive SPT/ATP. AAF was also provided (n = 51) to replace dairy (minimum 500 mL/day) whilst the remaining patients were prescribed a calcium supplement (n = 8). After 2 months, 47% had a normal biopsy when compared with pretreatment biopsies (*P* < .0009), median eosinophils reduced from 30 to 5/hpf (*P* < .0001) (Figure 2D) and 59% were in remission of EoE (defined as ≤5 eosinophils/hpf). However, only 30% of patients had ≥15 eosinophils/hpf (*P* < .0001) which was independent of AAF. The numbers assigned to receiving AAF and the calcium supplement were imbalanced in this study, which suggests that partial use of the ED is not as effective as exclusive ED. A similar 3‐month intervention which combined all three components of the EED, TED, and ED was studied by Kalach et al 2013^63^ in a retrospective cohort study of patients with EoE (n = 49), which found remission (defined as <5 eosinophils/hpf with no symptoms) in 53% of cases, as well as significant decreases in blood eosinophils (*P* < .0001), IgG (*P *= .003), and IgM (*P* < .05) levels. No other studies mentioned in this review measured serum levels of immunoglobulins.

### 3.3 Clinical symptoms

#### 3.3.1 ED only trials

Kelly et al 1994^53^ and 1995^54^ reported that after AAF use for 6 to 8 weeks, n = 8 patients had a resolution of clinical symptoms whilst the remaining had substantial improvements in chronic reflux. By continuing AAF, symptoms remained resolved for further 6 months (which included food refusal, profound disinterest in food, abdominal pain, vomiting, and diarrhea). Symptom improvement in this cohort while on AAF was reported as early as 3 weeks (median, 2‐6 weeks). Markowitz et al 2003^55^ (n = 51) found that the use of AAF led to a significant reduction in symptoms including vomiting, abdominal pain, dysphagia, heartburn, globus, water brash, and chest pain in all but two patients (*P *< .01). Those who responded to AAF did not differ to those who did not respond in terms of the type of symptom or degree of eosinophilia in this study.

#### 3.3.2 ED vs TED trials

Of the four studies that looked at ED and TED, only three reported clinical symptom change. Spergel et al 2005^57^ (n = 146) reported symptom resolution in 96% of the AAF group vs 78% from the TED group, however, the criteria outlining this was not stated. Those on AAF who demonstrated significant histological improvement (n = 160/164; 93%) also resolved their symptoms of reflux (n = 134 at baseline to n = 3 1‐month postdiet; *P *< .01) and dysphagia (n = 30 at baseline to n = 1 1‐month postdiet; *P *< .01). Furthermore, in those on AAF who had barium studies, normalization of esophageal narrowing was demonstrated in n = 21/22. However, n = 4 had no change in symptoms or eosinophilia despite compliance with AAF throughout the intervention. Liacouras et al 2005^62^ reported that of those in the TED group who demonstrated significant histological improvement (n = 75/132; 57%), the majority resolved their symptoms of reflux (n = 54 at baseline to n = 2 1‐month postdiet; *P *< .01) and dysphagia (n = 21 at baseline to n = 1 1‐month postdiet; *P *< .01). The effectiveness of the TED and AAF groups were not compared in this study. All patients who received AAF and were classified as “responders” in studies by Rizo Pascual et al 2011^56^ (n = 3) and Al‐Hussaini et al 2013^58^ (n = 3) were also identified as asymptomatic after the intervention. Symptoms before intervention included dysphagia, esophageal food impaction, vomiting, and abdominal pain.

#### 3.3.3 ED vs EED trials

Kagalwalla et al 2005^59^ (n = 60) found symptom response in 100% of patients on AAF who had either resolved (81%) or improved (11%) their symptoms, whereas only 95% of those on the EED had resolved or improved their symptoms whilst the remaining 5% had no change. In addition, complete mucosal healing was also observed in 56% of children in the AAF group.

#### 3.3.4 Combination intervention (ED, EED, and TED) trials

Colson et al 2014^61^ (n = 59) reported clinical improvements in 98% of digestive symptoms, 80% of cutaneous symptoms, 93% of respiratory symptoms and 100% of ear, nose, and throat symptoms after use of the combined EED, TED, and ED single intervention, although 30% were in non‐histological remission defined as ≥15 eosinophils/hpf. The difference in symptoms in those who took calcium supplements vs AAF was not analyzed. The only study that compared all three elimination diets did not report outcomes of symptom resolution.^60^

### 3.4 Growth

Of the 10 studies included in this review, only five authors reported growth outcomes. Kelly et al 1995^54^ (n = 10) reported that “poor weight gain had resolved” after intervention with AAF for 6 to 8 weeks and that “*each patient showed appropriate advancements of height and weight*,” but further information was not provided. Al‐Hussaini et al 2013^58^ reported that in those treated with AAF (n = 3) who were identified with failure to thrive prediet, growth corrected after 2‐month intervention. Liacouras et al 2005^62^ (n = 247) reported “no significant weight loss,” “or alteration of growth parameters (height, weight, and head circumference)” in those on dietary therapy, however, reported that n = 5 patients considered to have failure to thrive had a significant increase in weight after receiving AAF. Kagalwalla et al 2005^59^ (n = 60) reported that in the children with failure to thrive on AAF (n = 14) mean weight gain was 1.03 kg (range, 0.1‐2.1 kg), and of children identified with failure to thrive on the EED (n = 5), mean weight gain was 1.32 kg (range, 0.9‐2 kg), after 6 weeks of intervention. Colson et al 2014^61^ (n = 59) found that after use of the combined EED, TED, and ED single intervention, postdiet height, and weight gains were significant after 5 months, but weight‐for‐height *z*‐scores did not change.

### 3.5 Medications

Of the 10 studies included in this review, only one group of authors Kelly et al 1994^53^ and 1995^54^ investigated medication changes. From both cohorts it was found that 80%‐100% of patients discontinued their antireflux medications 6 months after intervention with AAF.

## 4 DISCUSSION

The effective use of AAF in the management of EoE as shown in this review implies there are several food allergens that may be implicated in esophageal inflammation which are not excluded from the diet when using the TED or the EED. The use of AAF ensures all food allergens are removed from the diet, which is more likely to induce remission and symptom resolution. As the EED and TED carry the risk of contamination and allergenic potential of unidentified dietary proteins, these diets may be rendered ineffective. Some data in this review suggests that the TED had the lowest remission response, which could be explained by the poor predictive value and clinical specificity of SPTs and APTs at guiding disease remission by food allergen. It is important to acknowledge that changes in histology or clinical symptoms observed in the trials on combination interventions where aspects of all three diets were used together^61^, ^63^ cannot be attributed to exclusive feeding with AAF. Adherence to any type of diet therapy and support given in each intervention was not compared or discussed in any of the studies. Nonresponsiveness of diet was not detected as an issue in any of the interventions with AAF, excluding one exceptional case where the child went onto to develop eosinophilic gastroenteritis.^57^ Overall 99.9%, (n = 461/462) of children with EoE responded to treatment with AAF.

Correlation between tissue eosinophil count and symptomatic remission or resolution did not necessarily match as demonstrated by Kagalwalla et al^59^ who found that although 88% of those on AAF had eosinophils ≤10/hpf, 100% had symptom resolution/improvement and similarly of those in the EED group, 74% had eosinophils ≤10/hpf, but 95% had symptom resolution/improvement. Colson et al^61^ also found that of 80%‐100% of pediatric EoE patients on a combined intervention with AAF, EED, and TED who demonstrated symptom improvement (digestive and respiratory), 30% exhibited eosinophils ≥15/hpf. On the contrary, it is known that in practice some patients have continued symptoms despite apparent normal eosinophilic histology, and it is recommended biopsies are taken at various levels of the esophagus.^10^ In this review, seven of the authors performed biopsies at various levels of the esophagus.^56^, ^57^, ^58^, ^59^, ^60^, ^61^, ^62^, ^63^ Overall, this emphasizes that symptoms and histology should be accurately monitored and interpreted together.

As nutritional intake, growth, and development are critical in children, including those with EoE, it was surprisingly very few studies assessed this, especially as restrictive diets, particularly in those with a food allergy, can impact on growth and nutritional deficiencies.^64^ In the studies that did, the use of AAF led to improvements at follow‐up. It is possible multi‐nutrient AAF provides all essential nutrients for patients with EoE, whereas the other diets may not be as nutritionally complete. Further research is needed to fully assess nutritional status, growth, and interventions in EoE such as AAF to recommend the frequency of monitoring of growth and other aspects of nutrition (eg, micronutrient status).

Discontinuation of PPIs was not studied in all of the trials included in this review, however, in one cohort the majority using AAF stopped PPIs after 6 months.^53^, ^54^ A recent meta‐analysis on the safety of long‐term PPI use in adults found an increased risk of fractures in those on short (<1 year) and long‐term PPI use which is believed to be related to reduced gastric acidity interfering with calcium absorption.^65^ AAF as management for pediatric EoE may help to alleviate prolonged PPI use, however, this needs to be examined further, especially as guidelines state that first‐line management of EoE with PPIs can be an effective therapy in the majority of patients.^10^

This review aimed to include all available literature investigating the use of AAF in pediatric patients with EoE, but the literature search method was not systematic or exhaustive, a meta‐analysis was not undertaken, and other forms of the diet in the management of pediatric EoE were not reviewed here. Although all the studies included provided positive outcomes for the use of AAF in pediatric EoE, most of the evidence comes from small quasi‐experimental, or retrospective cohort studies with imbalanced intervention groups, most likely due to the low feasibility of performing randomized controlled trials and the ethical constraints around with‐holding treatment in this relatively small and sensitive patient group. Many of the trials were conducted in specialist medical centers where the timing of repeat biopsies between intervention differed and could not be standardized due to study design (ie, retrospective). Population characteristics were heterogeneous across the studies with variations in age, differences in clinical history, differences in prokinetic, steroid or surgical history, and variation in PPI dosage. Definition of resolution was also different amongst some of the studies varying from ≤5 eosinophils/hpf^55^, ^57^, ^58^, ^61^ to ≤10 eosinophils/hpf^56^, ^59^ to ≤15 eosinophils/hpf.^60^ In addition there was a difference in the diagnosis of EoE based on eosinophilic count as some authors defined EoE as >20 eosinophils/hpf^55^, ^59^, ^62^ whereas others used >15 eosinophils/hpf which is supported by leading clinical guidelines.^57^, ^60^, ^61^ The assessment of symptoms was not systematic across all studies and no validated tools were used which does not omit the possibility of the placebo effect.

Although this review found that AAF is a clinically effective option for the dietary management of pediatric EoE, more robust research is required to understand how to effectively manage the diet in the long‐term in this chronic inflammatory condition. Specifically, establishing which patients are most likely to benefit from AAF and how the diet can be normalized soon after the resolution is imperative. There is little evidence on the negative psychosocial impact on feeding as a result of long‐term elimination diets in pediatric EoE but it has been recognized.^66^ Restrictions on oral intake could impact key stages of development in infants, that is, weaning. Some of the studies did not exclusively feed with AAF and modified the approach to allow some intake of solids (eg, apple and grapes) and water (nonsignificant sources of protein)^54^, ^55^, ^62^; which is recommended by the American Academy of Allergy, Asthma, and Immunology guidelines on EoE.^67^ However, none of the studies included explored the possibility of increasing the quantity and variety of foods received by a patient treated with ED, beyond the induction of EoE remission.

Many of the interventions with AAF included in this review involved enteral tube feeding (Table 1) due to reports of poor compliance, where the main factor was palatability. This was more challenging for older children expected to consume approximately >1.5 L, and as a result, influenced choice towards other dietary interventions.^58^ Across many studies, patients refused insertion of nasogastric enteral feeding tubes which affected uptake and compliance to the ED. Authors in one study were able to assign the ED via enteral feeding tubes by explaining the long‐term side effects of steroid use and chronic symptoms.^55^ Insertion of enteral feeding tubes may involve temporary psychosocial repercussions as well as increased resources. Many of the studies in this review were conducted on older formulations of AAF, whereas newer, more palatable varieties exist and are designed for older children. Furthermore, most interventions with ED were relatively short (<18 weeks) therefore we do not fully understand the impact of long‐term ED use.

This review highlighted a crucial lack of evidence in the literature of the time taken to return to a normal diet and the approaches used to obtain the successful reintroduction of food. This is absolutely necessary to guide best practice, as well as understanding the long‐term management of the dietary avoidance required after the reintroduction phase.^66^ Sequences for reintroduction have been outlined but vary greatly and consensus on whether biopsy frequency after single foods or groups of foods is required by further research.^22^ Authors from one trial in this review suggested categories of foods groups for introduction every 4 to 6 weeks followed by repeat biopsy and observation of clinical symptoms after introduction of four to five new foods.^56^ Whereas another group of authors permitted single food reintroductions if eosinophils were <15/hpf and resulted in an asymptomatic response with <15 eosinophils/hpf.^60^ If symptoms developed, patients were advised to discontinue the suspected food and began the next introduction ten to 14 days later. Average time to return to normal diet in this one trial was 2.1 (±1.8) years for those on the ED, 0.9 (±0.5) years for those on the EED and 1.6 (±1.7) years for those on the TED.^60^

Simpler and popular dietary approaches outlining elimination such as the “step‐up” diet require further evaluation to understand its effectiveness.^16^ Most importantly, research needs to define the criteria to establish when one type of diet should be used over another, which considers the severity of symptoms, growth, age, and previous food allergies.

## CONFLICT OF INTERESTS

KA, GH, and RJS are employees of Nutricia, Advanced Medical Nutrition, UK. CV has received honorariums from Danone, Abbott, Mead Johnson Nutritionals, Nestle, and DBV unrelated to this manuscript.
